# Transcription–Replication Coordination

**DOI:** 10.3390/life12010108

**Published:** 2022-01-13

**Authors:** Marco Saponaro

**Affiliations:** Transcription Associated Genome Instability Laboratory, Institute of Cancer and Genomic Sciences, University of Birmingham, Birmingham B15 2TT, UK; m.saponaro@bham.ac.uk

**Keywords:** transcription, DNA replication, genome instability, DNA damage, G-MiDS, transcription–replication collision

## Abstract

Transcription and replication are the two most essential processes that a cell does with its DNA: they allow cells to express the genomic content that is required for their functions and to create a perfect copy of this genomic information to pass on to the daughter cells. Nevertheless, these two processes are in a constant ambivalent relationship. When transcription and replication occupy the same regions, there is the possibility of conflicts between transcription and replication as transcription can impair DNA replication progression leading to increased DNA damage. Nevertheless, DNA replication origins are preferentially located in open chromatin next to actively transcribed regions, meaning that the possibility of conflicts is potentially an accepted incident for cells. Data in the literature point both towards the existence or not of coordination between these two processes to avoid the danger of collisions. Several reviews have been published on transcription–replication conflicts, but we focus here on the most recent findings that relate to how these two processes are coordinated in eukaryotes, considering advantages and disadvantages from coordination, how likely conflicts are at any given time, and which are their potential hotspots in the genome.

## 1. Complexity of the Transcription Process

**Figure 1:**
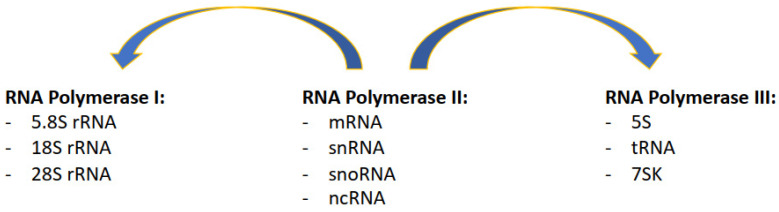
Description of which specific classes of transcripts are produced by the three RNA Polymerase complexes, with RNAPII supporting and also contributing to the transcription of RNAPI and RNAPIII transcripts.

Transcription is the process that produces RNA using DNA as a template. This allows cells to express the functional relevant parts of their genome and consents each cell in an organism to acquire specific functions. The diversity of transcripts that each cell can create permits the generation of 200 different cell types in the human body, all with virtually identical genomes. Importantly, this large variety of transcripts can be produced by different RNA Polymerase complexes, each responsible for a specific subset of transcripts (Figure 1):

RNA Polymerase I (RNAPI) transcribes the ribosomal RNA (rRNA 5.8S, 18S, and 28S in mammals), transcribed as a single polycistronic RNA subsequently processed in single rRNAs; ribosomal RNA transcripts are arranged in rDNA clusters present in the short arms of five human chromosomes (13, 14, 15, 21, and 22), in a broad range of copies of each unit per clusters [1].

RNA Polymerase II (RNAPII) transcribes messenger RNA (mRNA) from genes, approximately 42,000 in total in the human genome, half of which are protein coding and the other half noncoding [2]; RNAPII also transcribes long noncoding RNA (lncRNA), micro-RNA (miRNA), piwi-interacting RNA (piRNA), and most of the small nuclear RNA (snRNA) and small nucleolar RNA (snoRNA).

RNA Polymerase III (RNAPIII) transcribes transfer RNA (tRNA), with approximately 500 present in the human genome [3]; RNAPIII also transcribes the 5S rRNA that is arranged as a single cluster of approximately 100 repeats on chromosome 1 [1], and the remaining of the snRNA and snoRNA.

In reality, recent evidence has shown great crosstalk between the different complexes, meaning that the distinction between the roles of each complex is less neat than previously thought. For example, for a long time, it was known that RNAPII and RNAPII-associated transcription factors are present next to sites of RNAPIII transcription [4,5,6]. More recently, it was shown that RNAPII regulates the transcription of some RNAPIII transcripts [7]. In parallel, RNAPII-associated transcription factors regulate RNAPI subunits expression [8], and RNAPII is found at rDNA sites, essential to support ribosome biogenesis (Figure 1) [9]. Equally difficult is clearly determining which parts of the genome are transcribed. Protein coding genes, of which there are approximately 21,000 in the human genome and that represent the most varied category of transcribed regions, account for approximately 3% of the total genome [10]. Nevertheless, at least 75% of the genome can be transcribed, with most of the transcripts presenting features of RNAPII transcription [11]. It becomes immediately obvious that transcription is a totally pervasive process that can occupy most of the genome at the convergence point of many different cellular processes. As well as supporting each other’s transcription, with RNAPII involved in the transcription of all RNA polymerases, RNA polymerases can likewise conflict with each other. For example, the transcription of a gene can affect the ability to transcribe another transcript downstream, in a process known as transcription interference [12]; RNA polymerase complexes can collide with each other when converging, as eukaryotic RNA polymerases cannot bypass each other [13,14]. In this complex scenario, we have to consider that transcription is not the only process that uses the DNA as a substrate, as this is also used by DNA replication.

## 2. Transcription-Induced Genome Instability

RNA polymerases complexes have molecular weights of more than 500 kDa even without accessory transcription factors, and as such, are much bigger than the physical barriers that replicative helicases can overcome on DNA [15]. Consequently, conflicts between the transcription and replication machinery create a particularly dangerous situation, as impediments to replication forks progression can induce an increase in DNA damage and genome instability (a condition generally referred to as replication stress) [16,17]. Indeed, studies in vitro and in vivo from bacteria to eukaryotes have shown how head-to-head collisions are more detrimental than codirectional ones in interfering with replication fork progression [18,19,20,21,22,23,24]. Intriguingly, in the case of codirectional collisions, the replication machinery could take advantage of the mRNA present there to restart and continue replication after the collision site [18]. This finding is supported by in vivo data in bacteria with evidence of replication restart at codirectional collision sites [20]. However, an increase in codirectional collisions induced, for example, by an accumulation of backtracked RNAPII can be equally dangerous for genome stability, because the restart of replication downstream of the collision site can lead to an accumulation of single-strand gaps [25]. Altogether, these data indicate that a head-to-head or a codirectional collision between transcription and replication machinery could impact very differently on replication’s ability to progress with its task.

**Figure 2:**
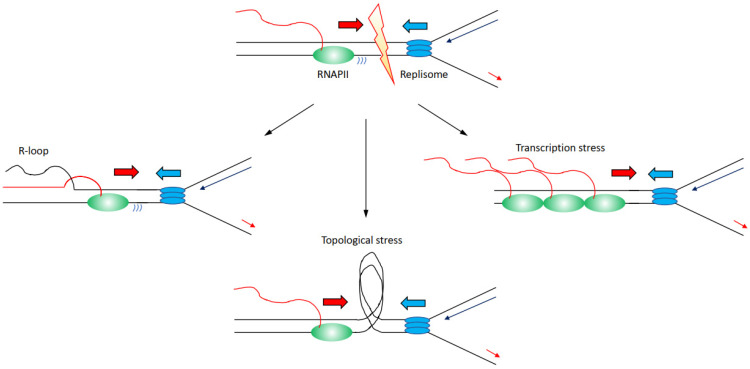
Description of the molecular mechanisms through which transcription has been identified to affect replication fork progression, inducing increased genome instability. Transcribing RNAPII is depicted as green oval with nascent RNA as a red line, replisome is depicted as blue ovals. Transcription can lead to (i) an accumulation of R-loops (left); (ii) an accumulation of topological constraints due to supercoilings generated by both transcription and replication (middle); (iii) an accumulation of stalled/paused/backtracked RNAPII known as transcription stress. For simplicity, transcription and replication have been presented in a head-to-head conformation, but this is not the unique condition that leads to increased genome instability.

There are several mechanisms through which transcription can affect directly or indirectly DNA replication progression inducing genome instability (Figure 2):

(i) Increased formation and/or persistence of three-stranded RNA–DNA hybrid structures called R-loops, formed when the nascent RNA hybridises back with the template DNA strand displacing the non-template DNA strand [26,27]; R-loops can furthermore lead to changes in chromatin structure and accessibility [28,29,30].

(ii) Accumulation of positive and negative supercoiling that induces increased topological constraints [31,32,33].

(iii) Accumulation of stalled/paused/backtracked RNA polymerase (so called transcription stress) [25,34].

(iv) Increased occurrence of DNA damages at transcribed regions [35,36,37].

Nevertheless, the distinction between these mechanisms is not neat, and for example, impairments of topoisomerases and transcription stress can likewise lead to increases in R-loops levels [32,38,39]. Altogether, large evidence indicates that transcription is a driver of genome instability, and in this sense, many RNAPII-associated factors are identified as essential to preserve genome stability [16,17,22]. Another evidence that links transcription to increased genome instability comes from the analyses of the genomic sites more prone to DNA damage when DNA replication is impaired. Common fragile sites (CFS) are chromosomal regions prone to breakage following low levels of replication stress, such as treatments with low doses of aphidicolin [40,41,42]. The propensity to genome instability of CFS is observed correspondingly in human diseases, with CFS identified as hotspots for genomic breakages in cancer cells, ultimately inducing the expression of oncogenes or deregulating the expression of oncosuppressors [43,44,45]. Several identified mechanisms explain CFS’s instability, among which there is also a paucity for replication origins and the fact the CFS are generally replicated later in S-phase [46,47,48,49]. Importantly, CFS are moreover enriched for long transcribed RNAPII genes, linking genome instability directly at CFS to RNAPII transcription [38]. The combination of both poor availability for replication origins with replication forks travelling long distances across large, transcribed domains, appears as the main determinants for genome instability at CFS [50]. Another class of fragile sites has been identified that is called early replicating fragile sites (ERFS) [51]. ERFS differ from CFS because ERFS accumulate breakages when cells are treated with high levels of replication stress induced by high doses of hydroxyurea, and ERFS breakages arise in early replicated regions [51]. ERFS overlap as CFS with genomic sites commonly lost in cancers and with transcribed genes, in the case of ERFS specifically with highly transcribed short genes [51]. Hence, as the data above indicate how transcribed regions are hotspots for genome instability, we must ask the question: how are transcription and DNA replication organised in order to avoid the dangerous consequences of conflicts and collisions?

## 3. Co-Existence or Spatial–Temporal Separation between Transcription and Replication

One simple possibility would be to keep the time when replication occurs in a cell separate from when the cell transcribes. It was known and thought for a long time that DNA replication is restricted exclusively to the S-phase of the cell cycle, and only once completed can cells progress into mitosis [52]; however, DNA synthesis can occur as late as in mitosis following treatments with replication stress-inducing agents, so-called mitotic DNA synthesis (MiDAS), in hotspot sites prone to replication stress such as CFS; nevertheless, there are a fraction of cells performing MiDAS even in the absence of exogenous replication stress treatments [53,54,55]. Regarding transcription’s regulation throughout the cell cycle, this is different depending on each specific RNA Polymerase. In the case of RNAPI, transcription levels oscillate throughout the cell cycle, with RNAPI transcription inactive only in mitosis and early G1 [56]. Consequently, during the replication of rDNA regions, ongoing replication forks and RNAPI transcription machinery are coordinated through the presence of specific replication fork barriers (RFB) present in each rDNA repeat unit [57,58]. The absence of functional RFB leads to collisions between the transcription and replication machinery [58], with many replication fork stability factors important to preserve rDNA repeat stability [59]. RNAPIII transcription activity is also low in early G1 and increases as cells progress through the cell cycle, becoming repressed in mitosis [60,61,62]. Indeed, it was shown in *S. cerevisiae* that tRNAs act as hotspots where replication fork stalls and pauses [63]. Finally, RNAPII is active at any stage of the cell cycle as RNAPII transcribes specific genes even in mitosis despite condensed chromosomes, although the vast majority of RNAPII complexes are allowed to complete transcription just before entering mitosis, with new initiation events inhibited [64,65]. Gene transcription levels are, however, not constant through the cell cycle, as many genes greatly change their levels depending on roles and functions. Even so, many genes are specifically upregulated or expressed during the S-phase, for example, components of the replication machinery and histones required to pack the newly replicated DNA into chromatin [66]. Considering all data together, during the S-phase, all three RNA Polymerases are active, indicating that the timely separation of transcription and replication is not a strategy that eukaryotic cells deploy to avoid the occurrence of collisions.

An additional layer of complexity comes from the analysis of DNA replication timing and the distribution of replication origins. Several studies across many model systems have invariably shown that transcribed regions are preferentially replicated in the early S-phase, while poorly transcribed regions are preferentially replicated in the late S-phase [67,68,69]. Consequently, as different cell types will transcribe distinct regions of their genome depending on their role and function, there is not a unique replication program in higher eukaryotes, as this will be cell type specific and affected by which regions a specific cell transcribes [69]. Moreover, considering the diverse transcription programs that different cell types have, it would be virtually impossible to have replication origins activated so that replication forks would be uniquely codirectional with highly transcribed genes to avoid more challenging head-to-head collisions, as it happens in bacteria [57,69]. Finally, mapping of DNA replication origins has shown that these are enriched next to the transcription start sites (TSSs) of actively transcribed genes, colocalising with active histone marks [70,71,72,73]. Importantly, replication origins are preferentially enriched near TSSs of long genes, arranged so that the leading replication fork and RNAPII are codirectional [73]. This set of evidence would suggest that cells do not or cannot arrange replication origins to completely avoid potential encounters between transcription and replication. If anything, by arranging leading replication forks to be codirectional with transcription along long genes, the replication program is pre-emptying potentially more troublesome instances such as head-to-head collisions. Actually, there could be benefits for cells in activating replication origins next to transcribed sites, as the open chromatin conformation of transcribed regions makes them more accessible for the replication machinery too. Moreover, by starting replication of the genome from the transcribed regions, cells make sure that they will pass to their daughter cells the genetic information that is needed for their function; therefore, is there evidence supporting the existence of a higher level organisation that coordinates transcription and replication? Are cells actively controlling these two processes to reduce the risk of conflicts and ultimately preserve genome stability?

Some evidence in support of such an arrangement comes from studies analysing the nuclear distribution of active replication and active transcription throughout the S-phase. Some data show that transcription and replication occur in different parts of the nucleus throughout the S-phase, suggesting that when a region is replicated, it is not at the same time also transcribed [74]; however, other data show overlaps between transcription and replication, in particular, in early the S-phase [75]. The dissimilarity between these results could not be more striking, and even considering the technical differences between these papers in terms of labelling time or cell types, these findings do not answer whether transcription and replication are coordinated. Equally, genomic analyses assessing transcription and replication activities and dynamics throughout the S-phase have reached contrasting conclusions. Some data, for example, support segregation and temporal separation between transcription and replication: transcription levels and replication timings are inversely correlating, with early replicated genes increasing their transcription later during the S-phase, while late replicated genes reduce their transcription during the S-phase [76]. At the same time, others have identified that TSSs of actively transcribed genes maintain high levels of nascent transcription activity even when genes are replicated [77]. This transcription activity footprint at TSSs affects the replication of TSSs compared to the rest of the gene [77]. The reduced replication of TSSs persists throughout the cell cycle until G2/M, when the RNAPII is removed from most of the transcribed genes allowing the completion of the duplication of TSSs [77]. Hundreds of genes present DNA synthesis at TSSs, specifically in G2/M, especially genes characterised by high levels of TSS-associated antisense transcription [77]. This process is distinct from MiDAS, it is not associated with sites of DNA damage nor dependent on canonical DNA damage repair and response factors, and is called G2/M DNA synthesis (G-MiDS) [77]. TSSs have been further identified as hotspots of transcription replication interaction (TRI) zones in mouse cells [78]. Further, in this case, TRI zones are a relatively common and general instance with more than a thousand TSSs identified, in particular those characterised by the presence of transcription going in both directions, either because of bidirectional promoters or because of the presence of an annotated transcript [78]. Both the *Wang* et al. and the *St Germain* et al. papers show that hotspots of G-MiDS and TRI correlate with genomic sites frequently rearranged and mutated in tumours, linking once more transcription and replication conflict regions to hotspot sites of genome instability linked to human diseases [77,78]. Altogether, therefore, in the literature, there is both evidence supporting the existence of coordination between transcription and replication, as well as evidence that supports that the two processes coexist all the time together.

## 4. Pros and Cons from Coordinating Transcription and Replication

**Figure 3:**
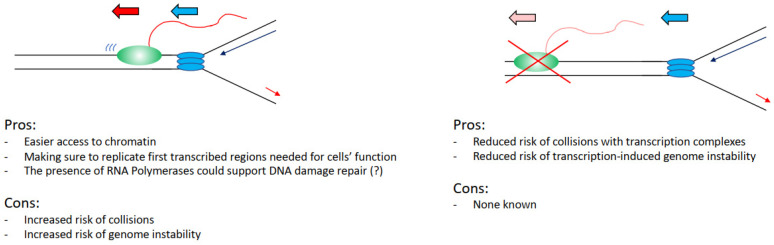
List of the advantages and the disadvantages that cells have in keeping transcription and replication spatially and temporally separated. Transcribing RNAPII is depicted as green oval with nascent RNA as a red line, replisome is depicted as blue ovals.

Can we, therefore, evaluate this problem from an evolutionary point of view, assessing perhaps what would be the best alternative for a cell in an ideal scenario? Practically analysing this situation, it would be convenient for a cell to keep the two processes separated, as interference between transcription and replication has been widely associated with increased DNA damage and genome instability. In support of this view, we have the fact that transcriptional defects drive directly increased DNA damage in cells [26,34], many transcription factors are found important to preserve genome stability [27], and the fact that fragile sites overlap with transcribed regions (Figure 3) [49,51]. Even the recent findings that show that transcription and replication coexist all the time emphasise how hotspot sites of interference overlap with genome instability sites in cancers [77,78].

However, again analysing the evidence hypothetically, there are benefits for a cell in having the two processes coexisting. It would be easier to load and activate replication initiation complexes in the open chromatin conformation of transcribed regions (Figure 3) [70,71,72,73]. Moreover, even in case of DNA damage arising from a collision event, there is the potential direct positive impact and contribution of transcription and transcription-associated chromatin modifications to the DNA damage repair kinetics and repair pathway choices. For example, in the case of nucleotide excision repair, the DNA damage-affected RNAPII can directly recruit and activate the transcription-coupled nucleotide excision repair sub-pathway, with faster DNA damage repair kinetics in transcribed regions than in not transcribed regions [79,80]. In the case of double-strand breaks (DSBs), these are preferentially repaired by homologous recombination instead of non-homologous end joining in transcribed regions, with non-homologous end joining preferentially repairing DSBs in not transcribed regions [81,82]. Moreover, in recent years it has become evident that RNA polymerases and transcription play a direct role in the correct establishment of DNA damage repair and response foci and the repair of DSBs. More specifically, RNAs produced at DSBs sites are required for the correct assembly of 53BP1 foci and for the formation of a phase separation state, important for the activation of the DNA damage response [83,84,85,86]. DSB induced RNAs are MRE11-dependent and their processing requires the DROSHA and DICER RNases involved in RNA interference [84,87,88]. An important step in this process is the generation of R-loops that facilitates the repair through RAD51-dependent homologous recombination, with many factors identified as important for R-loops establishment as well as R-loops resolution [89,90,91,92,93,94,95,96]. While currently, there is no evidence supporting that transcription could be directly involved in the resolution of transcription–replication collisions, a potential role for transcription cannot be completely excluded considering the above-mentioned data.

## 5. How Likely Are Transcription–Replication Collisions in a Cell

Following on from the above speculation, there appear to be more advantages in not coordinating transcription and replication than in coordinating the two processes. Perhaps a clearer evaluation of this conundrum can come from assessing how many transcripts are produced by the transcription machinery at any given time, and the broader impact of transcription on genome biology. As mentioned at the beginning, the three RNA polymerases are together responsible for transcribing thousands of different transcripts. Moreover, it is also clear that most of the transcription in terms of the number of different transcripts is performed by RNAPII, which also has its transcripts interspersed in the whole genome [11]. However, single cell analyses can identify only a few thousand mRNA transcripts per cell out of all the tens of thousands present in the genome [97]. Even more strikingly, the median number of mRNA molecules for each gene in a cell is only 17 mRNA molecules [97]. This number of mRNA molecules in the cell is the combination of molecules of mRNA synthesised and molecules of mRNA degraded; however, as the median half-life of mRNAs is of several hours, once the mRNA has been produced, it will be available for a considerable amount of time [97]. An important feature of transcription in higher eukaryotes is the fact that genes are transcribed in bursts of transcription, switching between ‘on’ and ‘off’ stages [98,99]. The lengths of the bursts and the number of mRNA molecules produced by each burst vary greatly from gene to gene and from cell to cell, affected by histone modifications and the composition of the pre-initiation complex [98,99,100,101]. Nevertheless, each ‘on’ burst can produce up to teens of mRNA copies. Hence, considering the above-mentioned mRNA half-life [98], it could practically take just a single transcriptional burst to produce all the mRNA molecules present for an “average” gene. Considering that the average median length of a human gene is approximately 27 kb [102] and that the average transcription elongation rate has been measured at 2–4 kb/min [34,103,104,105], it means that it will take a single RNAPII 7–14 min to transcribe the whole gene and produce one mRNA molecule. Following this first RNAPII, other RNAPIIs could trail in a transcription burst quickly, producing all the mRNA molecules present in the cell. Accordingly, at any given time, there will be only a very low number of RNAPII complexes in the gene body.

What these numbers indicate is that perhaps the two processes of transcription and replication do not need to be coordinated, as the low number of RNAPII complexes present in a gene by itself reduces the risk of a collision at any given time. Moreover, they can explain the contrasting findings mentioned earlier, such as the overlap and coordination data obtained by immunofluorescence and genome-wide analyses [74,75,76,77,78]: transcription and replication could coexist all the time and perform their tasks without coordinating with each other, as the likelihood of the overlap between them, and consequently the possibility of a conflict, is low.

**Figure 4:**
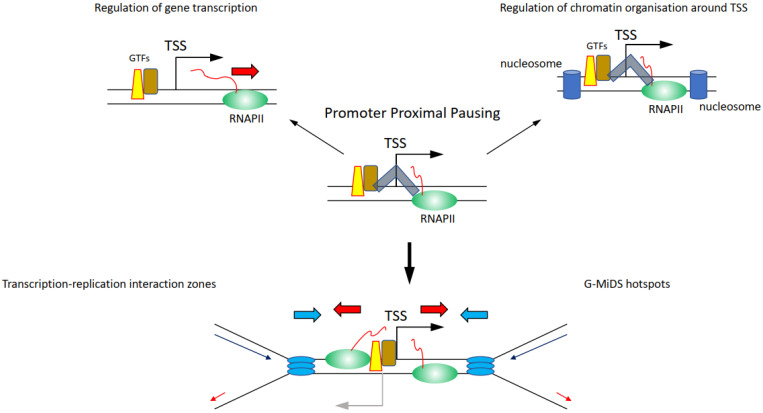
Impact and relevance of how regulation of transcription elongation, and in particular promoter-proximal pausing (PPP), is important to finely regulate transcription activity and chromatin organisation around the TSS. The DSIF-NELF complex (depicted as grey shape) is the crucial component in the PPP that maintains RNAPII near the TSS interacting with the general transcription factors (GTF) on the promoter. Following CDK9-dependent regulation of the DSIF-NELF complex, the RNAPII is released from PPP and can transcribe the gene (top left); at the same time, maintaining the RNAPII near the TSS is important to maintain the nucleosome (blue blocks) organisation around the TSS (top right). The consequence of upholding the RNAPII near the TSS means that replication forks are more likely to conflict and collide with RNAPII at the TSS (bottom). This is particularly the case at sites characterised by transcription going in both directions, hotspots for transcription–replication interaction zones and G-MiDS (below).

Whether components of the transcription machinery will remain on chromatin between ‘on’ bursts is not clear. If so, it is the promoter region that could represent a hotspot for high-risk transcription–replication conflicts, because of the high density of proteins present there (Figure 4) [106,107]. Among the proteins persisting at promoters, there is also the actual RNAPII, as the transition from initiation to elongation is a highly controlled process, meaning that even when RNAPII starts transcribing a gene, it can be halted at multiple points after the TSS. Promoter proximal pausing (PPP) halts the RNAPII 20–50 bp downstream of the TSS (Figure 4) [108]. This process was first identified on heat shock responsive genes and was proposed as a quick response mechanism to induce gene transcription following heat shock, simply by releasing the RNAPII from its promoter [108]. It was later found that PPP is a much more general transcription regulatory process controlled by CDK9 through the regulation of the NELF and the DSIF complexes [108,109]. Maintaining the RNAPII near the TSS through the PPP is moreover important to preserve the nucleosome organisation at TSSs, to protect the nucleosome-free region, and thus retain the ability to transcribe the gene (Figure 4) [110,111]. RNAPII is also controlled by CDK9 further downstream of the TSS and at the transcription termination site, although it is not clear what these regulatory steps are important for and whether they affect the replication of genes [34,112]. Intriguingly, knockdown of components of the NELF and DSIF complexes important to regulate PPP reduces RNAPII persistence at TSSs and G-MiDS frequency and levels, linking RNAPII occupancy at TSS to late cell cycle DNA synthesis (Figure 4) [77]. These data support that TSS surroundings are the hotspot sites where the transcription and replication machinery interact together and identify TSSs as the regions where to seek answers on how transcription and replication coordination takes place (Figure 4) [77].

Perhaps here is a key aspect in understanding the relationship between transcription and replication: cells need to maintain RNAPII near the TSS to maintain the correct chromatin structure of TSSs that will allow them to transcribe genes when needed. This chromatin organisation is also highly conserved during DNA replication, with nucleosome positioning maintained around the TSS also in newly replicated chromatin [113]. Altogether, maintaining RNAPII near TSSs would ultimately allow cells to create two fully functional copies of their genomes to pass on to the daughter cells.

It has been long known that TSSs and promoters represent hotspot sites of genome instability and DNA damage [114,115,116]. The immediate surrounding of the TSS is where RNAPII and transcription factors accumulate, as mentioned above, but TSSs are also sites where physiologic R-loops accumulate [117]. However, R-loops are also concentrated at the transcription termination sites and these are generally not identified as genome instability hotspots [114,115,116,117]. R-loops at TSSs play important roles in regulating transcription activity, recruiting TIP60 to acetylate histones and maintain gene transcription [118], and to preserve TSS-associated antisense transcription [119]. Considering also that R-loops formation and turnover at TSSs is highly dynamic and follows genes’ transcription activity [120], it is unlikely that the presence of R-loops at TSSs is the source of the conflicts between transcription and replication at these sites and the main reason for the increased genome instability there.

## 6. Concluding Remarks

In the future, it will be important to determine what factors and pathways are involved in the coordination between transcription and replication when these two processes are close together, but also defining whether there is an increased risk of genome instability every time transcription and replication collide. This may depend on many factors such as on the directionality between transcription and replication, affected by the local chromatin environment and histone marks, the RNAPII status, or ultimately whether these collisions will impair the ability to progress of either the RNAPII or the replisome.

## References

[1] Stults D.M., Killen M.W., Pierce H.H., Pierce A.J. (2008). Genomic architecture and inheritance of human ribosomal RNA gene clusters. Genome Biol..

[2] Pertea M., Shumate A., Pertea G., Varabyou A., Breitwieser F.P., Chang Y.C., Madugundu A.K., Pandey A., Salzberg S.L. (2018). CHESS: A new human gene catalog curated from thousands of large-scale RNA sequencing experiments reveals extensive transcriptional noise. Genome Biol..

[3] Dieci G., Fiorino G., Castelnuovo M., Teichmann M., Pagano A. (2007). The expanding RNA polymerase III transcriptome. Trends Genet..

[4] Barski A., Chepelev I., Liko D., Cuddapah S., Fleming A.B., Birch J., Cui K., White R.J., Zhao K. (2010). Pol II and its associated epigenetic marks are present at Pol III-transcribed noncoding RNA genes. Nat. Struct. Mol. Biol..

[5] Canella D., Praz V., Reina J.H., Cousin P., Hernandez N. (2010). Defining the RNA polymerase III transcriptome: Genome-wide localization of the RNA polymerase III transcription machinery in human cells. Genome Res..

[6] Raha D., Wang Z., Moqtaderi Z., Wu L., Zhong G., Gerstein M., Struhl K., Snyder M. (2010). Close association of RNA polymerase II and many transcription factors with Pol III genes. Proc. Natl. Acad. Sci. USA.

[7] Gerber A., Ito K., Chu C.S., Roeder R.G. (2020). Gene-Specific control of tRNA expression by RNA Polymerase II. Mol. Cell.

[8] Bywater M.J., Poortinga G., Sanij E., Hein N., Peck A., Cullinane C., Wall M., Cluse L., Drygin D., Anderes K. (2012). Inhibition of RNA Polymerase I as a therapeutic strategy to promote cancer-specific activation of p53. Cancer Cell.

[9] Abraham K.J., Khosraviani N., Chan J.N.Y., Gorthi A., Samman A., Zhao D.Y., Wang M., Bokros M., Vidya E., Ostrowski L.A. (2020). Nucleolar RNA polymerase II drives ribosome biogenesis. Nature.

[10] Pennisi E. (2012). Genomics. ENCODE project writes eulogy for junk DNA. Science.

[11] Djebali S., Davis C.A., Merkel A., Dobin A., Lassmann T., Mortazavi A., Tanzer A., Lagarde J., Lin W., Schlesinger F. (2012). Landscape of transcription in human cells. Nature.

[12] Proudfoot N.J. (1986). Transcriptional interference and termination between duplicated alpha-globin gene constructs suggests a novel mechanism for gene regulation. Nature.

[13] Prescott E.M., Proudfoot N.J. (2002). Transcriptional collision between convergent genes in budding yeast. Proc. Natl. Acad. Sci. USA.

[14] Hobson D.J., Wei W., Steinmetz L.M., Svejstrup J.Q. (2012). RNA polymerase II collision interrupts convergent transcription. Mol. Cell.

[15] Yardimci H., Wang X., Loveland A.B., Tappin I., Rudner D.Z., Hurwitz J., van Oijen A.M., Walter J.C. (2012). Bypass of a protein barrier by a replicative DNA helicase. Nature.

[16] Hamperl S., Cimprich K.A. (2016). Conflict resolution in the genome: How transcription and replication make it work. Cell.

[17] Gomez-Gonzalez B., Aguilera A. (2019). Transcription-mediated replication hindrance: A major driver of genome instability. Genes Dev..

[18] Pomerantz R.T., O’Donnell M. (2008). The replisome uses mRNA as a primer after colliding with RNA polymerase. Nature.

[19] Pomerantz R.T., O’Donnell M. (2010). Direct restart of a replication fork stalled by a head-on RNA polymerase. Science.

[20] Merrikh H., Machón C., Grainger W.H., Grossman A.D., Soultanas P. (2011). Co-directional replication-transcription conflicts lead to replication restart. Nature.

[21] Prado F., Aguilera A. (2005). Impairment of replication fork progression mediates RNA polII transcription-associated recombination. EMBO J..

[22] Hamperl S., Bocek M.J., Saldivar J.C., Swigut T., Cimprich K.A. (2017). Transcription-replication conflict orientation modulates R-Loop levels and activates distinct DNA damage responses. Cell.

[23] Wang J.D., Berkman M.B., Grossman A.D. (2007). Genome-wide coorientation of replication and transcription reduces adverse effects on replication in Bacillus subtilis. Proc. Natl. Acad. Sci. USA.

[24] Mirkin E.V., Mirkin S.M. (2005). Mechanisms of transcription-replication collisions in bacteria. Mol. Cell Biol..

[25] Dutta D., Shatalin K., Epshtein V., Gottesman M.E., Nudler E. (2011). Linking RNA polymerase backtracking to genome instability in *E. coli.*. Cell.

[26] Huertas P., Aguilera A. (2003). Cotranscriptionally formed DNA:RNA hybrids mediate transcription elongation impairment and transcription-associated recombination. Mol. Cell.

[27] Paulsen R.D., Soni D.V., Wollman R., Hahn A.T., Yee M.C., Guan A., Hesley J.A., Miller S.C., Cromwell E.F., Solow-Cordero D.E. (2009). A genome-wide siRNA screen reveals diverse cellular processes and pathways that mediate genome stability. Mol. Cell.

[28] Skourti-Stathaki K., Kamieniarz-Gdula K., Proudfoot N.J. (2014). R-loops induce repressive chromatin marks over mammalian gene terminators. Nature.

[29] Castellano-Pozo M., Santos-Pereira J.M., Rondón A.G., Barroso S., Andújar E., Pérez-Alegre M., García-Muse T., Aguilera A. (2013). R loops are linked to histone H3 S10 phosphorylation and chromatin condensation. Mol. Cell.

[30] Groh M., Lufino M.M., Wade-Martins R., Gromak N. (2014). R-loops associated with triplet repeat expansions promote gene silencing in Friedreich ataxia and fragile X syndrome. PLOS Genet..

[31] Bermejo R., Capra T., Gonzalez-Huici V., Fachinetti D., Cocito A., Natoli G., Katou Y., Mori H., Kurokawa K., Shirahige K. (2009). Genome-organizing factors Top2 and Hmo1 prevent chromosome fragility at sites of S phase transcription. Cell.

[32] Tuduri S., Crabbé L., Conti C., Tourrière H., Holtgreve-Grez H., Jauch A., Pantesco V., De Vos J., Thomas A., Theillet C. (2009). Topoisomerase I suppresses genomic instability by preventing interference between replication and transcription. Nat. Cell Biol..

[33] Promonet A., Padioleau I., Liu Y., Sanz L., Biernacka A., Schmitz A.L., Skrzypczak M., Sarrazin A., Mettling C., Rowicka M. (2020). Topoisomerase 1 prevents replication stress at R-loop-enriched transcription termination sites. Nat. Commun..

[34] Saponaro M., Kantidakis T., Mitter R., Kelly G.P., Heron M., Williams H., Söding J., Stewart A., Svejstrup J.Q. (2014). RECQL5 controls transcript elongation and suppresses genome instability associated with transcription stress. Cell.

[35] Poetch A.R., Boulton S.J., Luscombe N.M. (2018). Genomic landscape of oxidative DNA damage and repair reveals regioselective protection from mutagenesis. Genome Biol..

[36] Wu W., Hill S.E., Nathan W.J., Paiano J., Callen E., Wang D., Shinoda K., van Wietmarschen N., Colón-Mercado J.M., Zong D. (2021). Neuronal enhancers are hotspots for DNA single-strand break repair. Nature.

[37] Jiang Y., Li W., Lindsey-Boltz L.A., Yang Y., Li Y., Sancar A. (2021). Super hotspots and super coldspots in the repair of UV-induced DNA damage in the human genome. JBC.

[38] Marinello J., Bertoncini S., Aloisi I., Cristini A., Malagoli Tagliazucchi G., Forcato M., Sordet O., Capranico G. (2016). Dynamic effects of topoisomerase I inhibition on R-Loops and short transcripts at active promoters. PLoS ONE.

[39] Zatreanu D., Han Z., Mitter R., Tumini E., Williams H., Gregersen L., Dirac-Svejstrup A.B., Roma S., Stewart A., Aguilera A. (2019). Elongation factor TFIIS prevents transcription stress and R-Loop accumulation to maintain genome stability. Mol. Cell.

[40] Glover T.W., Berger C., Coyle J., Echo B. (1984). DNA polymerase alpha inhibition by aphidicolin induces gaps and breaks at common fragile sites in human chromosomes. Hum. Genet..

[41] Debacker K., Frank Kooy R. (2007). Fragile sites and human disease. Hum. Mol. Genet..

[42] Debatisse M., Le Tallec B., Letessier A., Dutrillaux B., Brison O. (2012). Common fragile sites: Mechanisms of instability revisited. Trends Genet..

[43] Bignell G.R., Greenman C.D., Davies H., Butler A.P., Edkins S., Andrews J.M., Buck G., Chen L., Beare D., Latimer C. (2010). Signatures of mutation and selection in the cancer genome. Nature.

[44] Hazan I., Hofman T.G., Aqeilan R.i. (2016). Tumor suppressor genes within common fragile sites are active players in the DNA damage response. PLOS Genet..

[45] Casper A.M., Nghiem P., Arlt M.F., Glover T.W. (2002). ATR regulates fragile site stability. Cell.

[46] Pirzio L.M., Pichierri P., Bignami M., Franchitto A. (2008). Werner syndrome helicase activity is essential in maintaining fragile site stability. J. Cell Biol..

[47] Letessier A., Millot G.A., Koundrioukoff S., Lachagès A.M., Vogt N., Hansen R.S., Malfoy B., Brison O., Debatisse M. (2011). Cell-type-specific replication initiation programs set fragility of the FRA3B fragile site. Nature.

[48] Bergoglio V., Boyer A.S., Walsh E., Naim V., Legube G., Lee M.Y., Rey L., Rosselli F., Cazaux C., Eckert K.A. (2013). DNA synthesis by Pol η promotes fragile site stability by preventing under-replicated DNA in mitosis. J. Cell Biol..

[49] Le Tallec B., Millot G.A., Blin M.E., Brison O., Dutrillaux B., Debatisse M. (2013). Common fragile site profiling in epithelial and erythroid cells reveals that most recurrent cancer deletions lie in fragile sites hosting large genes. Cell Rep..

[50] Brison O., El-Hilali S., Azar D., Koundrioukoff S., Schmidt M., Nähse V., Jaszczyszyn Y., Lachages A.M., Dutrillaux B., Thermes C. (2019). Transcription-mediated organization of the replication initiation program across large genes sets common fragile sites genome-wide. Nat. Commun..

[51] Barlow J.H., Faryabi R.B., Callén E., Wong N., Malhowski A., Chen H.T., Gutierrez-Cruz G., Sun H.W., McKinnon P., Wright G. (2013). Identification of early replicating fragile sites that contribute to genome instability. Cell.

[52] Howard A., Pelc S.R. (1953). Synthesis of deoxyribonucleic acid in normal and irradiated cells and its relation to chromosome breakage. Heredity.

[53] Minocherhomji S., Ying S., Bjerregaard V.A., Bursomanno S., Aleliunaite A., Wu W., Mankouri H.W., Shen H., Liu Y., Hickson I.D. (2015). Replication stress activates DNA repair synthesis in mitosis. Nature.

[54] Bhowmick R., Minocherhomji S., Hickson I.D. (2016). RAD52 facilitates mitotic DNA synthesis following replication stress. Mol. Cell.

[55] Maya-Mendoza A., Moudry P., Merchut-Maya J.M., Lee M., Strauss R., Bartek J. (2018). High speed of fork progression induces DNA replication stress and genomic instability. Nature.

[56] Klein J., Grummt I. (1999). Cell cycle-dependent regulation of RNA polymerase I transcription: The nucleolar transcription factor UBF is inactive in mitosis and early G1. Proc. Natl. Acad. Sci. USA.

[57] Brewer B.J., Fangman W.L. (1988). A replication fork barrier at the 3′ end of yeast ribosomal RNA genes. Cell.

[58] Akamatsu Y., Kobayashi T. (2015). The human RNA Polymerase I transcription terminator complex acts as a replication fork barrier that coordinates the progress of replication with rRNA transcription activity. Mol. Cell Biol..

[59] Warmerdam D.O., Wolthuis R.M.F. (2019). Keeping ribosomal DNA intact: A repeating challenge. Chromosome Res..

[60] White R.J., Gottlieb T.M., Downes C.S., Jackson S.P. (1995). Cell cycle regulation of RNA polymerase III transcription. Mol. Cell Biol..

[61] Scott P.H., Cairns C.A., Sutcliffe J.E., Alzuherri H.M., McLees A., Winter A.G., White R.J. (2001). Regulation of RNA polymerase III transcription during cell cycle entry. JBC.

[62] Gottesfeld J.M., Wolf V.J., Dang T., Forbes D.J., Hartl P. (1994). Mitotic repression of RNA polymerase III transcription in vitro mediated by phosphorylation of a TFIIIB component. Science.

[63] Azvolinsky A., Giresi P.G., Lieb J.D., Zakian V.A. (2009). Highly transcribed RNA polymerase II genes are impediments to replication fork progression in Saccharomyces cerevisiae. Mol. Cell.

[64] Liang K., Woodfin A.R., Slaughter B.D., Unruh J.R., Box A.C., Rickels R.A., Gao X., Haug J.S., Jaspersen S.L., Shilatifard A. (2015). Mitotic transcriptional activation: Clearance of actively engaged Pol II via transcriptional elongation control in mitosis. Mol. Cell.

[65] Palozola K.C., Donahue G., Liu H., Grant G.R., Becker J.S., Cote A., Yu H., Raj A., Zaret K.S. (2017). Mitotic transcription and waves of gene reactivation during mitotic exit. Science.

[66] van der Meijden C.M., Lapointe D.S., Luong M.X., Peric-Hupkes D., Cho B., Stein J.L., van Wijnen A.J., Stein G.S. (2002). Gene profiling of cell cycle progression through S-phase reveals sequential expression of genes required for DNA replication and nucleosome assembly. Cancer Res..

[67] Schübeler D., Scalzo D., Kooperberg C., van Steensel B., Delrow J., Groudine M. (2002). Genome-wide DNA replication profile for Drosophila melanogaster: A link between transcription and replication timing. Nat. Genet..

[68] Woodfine K., Fiegler H., Beare D.M., Collins J.E., McCann O.T., Young B.D., Debernardi S., Mott R., Dunham I., Carter N.P. (2004). Replication timing of the human genome. Hum. Mol. Genet..

[69] Hansen R.S., Thomas S., Sandstrom R., Canfield T.K., Thurman R.E., Weaver M., Dorschner M.O., Gartler S.M., Stamatoyannopoulos J.A. (2010). Sequencing newly replicated DNA reveals widespread plasticity in human replication timing. Proc. Natl. Acad. Sci. USA.

[70] Karnani N., Taylor C.M., Malhotra A., Dutta A. (2010). Genomic study of replication initiation in human chromosomes reveals the influence of transcription regulation and chromatin structure on origin selection. Mol. Biol. Cell.

[71] Dellino G.I., Cittaro D., Piccioni R., Luzi L., Banfi S., Segalla S., Cesaroni M., Mendoza-Maldonado R., Giacca M., Pelicci P.G. (2013). Genome-wide mapping of human DNA-replication origins: Levels of transcription at ORC1 sites regulate origin selection and replication timing. Genome Res..

[72] Petryk N., Kahli M., d’Aubenton-Carafa Y., Jaszczyszyn Y., Shen Y., Silvain M., Thermes C., Chen C.L., Hyrien O. (2016). Replication landscape of the human genome. Nat. Commun..

[73] Chen Y.H., Keegan S., Kahli M., Tonzi P., Fenyö D., Huang T.T., Smith D.J. (2019). Transcription shapes DNA replication initiation and termination in human cells. Nat. Struct. Mol. Biol..

[74] Wei X., Samarabandu J., Devdhar R.S., Siegel A.J., Acharya R., Berezney R. (1998). Segregation of transcription and replication sites into higher order domains. Science.

[75] Hassan A.B., Errington R.J., White N.S., Jackson D.A., Cook P.R. (1994). Replication and transcription sites are colocalized in human cells. J. Cell Sci..

[76] Meryet-Figuiere M., Alaei-Mahabadi B., Ali M.M., Mitra S., Subhash S., Pandey G.K., Larsson E., Kanduri C. (2014). Temporal separation of replication and transcription during S-phase progression. Cell Cycle.

[77] Wang J., Rojas P., Mao J., Mustè Sadurnì M., Garnier O., Xiao S., Higgs M.R., Garcia P., Saponaro M. (2021). Persistence of RNA transcription during DNA replication delays duplication of transcription start sites until G2/M. Cell Rep..

[78] St Germain C.P., Zhao H., Sinha V., Sanz L.A., Chedin F., Barlow J.H. (2021). Genomic patterns of transcription-replication interactions in mouse primary B cells. Biorxiv.

[79] Adar S., Hu J., Lieb J.D., Sancar A. (2016). Genome-wide kinetics of DNA excision repair in relation to chromatin state and mutagenesis. Proc. Natl. Acad. Sci. USA.

[80] Marteijn J.A., Lans H., Vermeulen W., Hoeijmakers J.H. (2014). Understanding nucleotide excision repair and its roles in cancer and ageing. Nat. Rev. Cell Mol. Biol..

[81] Aymard F., Bugler B., Schmidt C.K., Guillou E., Caron P., Briois S., Iacovoni J.S., Daburon V., Miller K.M., Jackson S.P. (2014). Transcriptionally active chromatin recruits homologous recombination at DNA double-strand breaks. Nat. Struct. Mol. Biol..

[82] Clouaire T., Rocher V., Lashgari A., Arnould C., Aguirrebengoa M., Biernacka A., Skrzypczak M., Aymard F., Fongang B., Dojer N. (2018). Comprehensive mapping of histone modifications at DNA double-strand breaks deciphers repair pathway chromatin signatures. Mol. Cell.

[83] Pryde F., Khalili S., Robertson K., Selfridge J., Ritchie A.M., Melton D.W., Jullien D., Adachi Y. (2005). 53BP1 exchanges slowly at the sites of DNA damage and appears to require RNA for its association with chromatin. J. Cell Sci..

[84] Francia S., Michelini F., Saxena A., Tang D., de Hoon M., Anelli V., Mione M., Carninci P., d’Adda di Fagagna F. (2012). Site-specific DICER and DROSHA RNA products control the DNA-damage response. Nature.

[85] Pessina F., Giavazzi F., Yin Y., Gioia U., Vitelli V., Galbiati A., Barozzi S., Garre M., Oldani A., Flaus A. (2019). Functional transcription promoters at DNA double-strand breaks mediate RNA-driven phase separation of damage-response factors. Nat. Cell Biol..

[86] Kilic S., Lezaja A., Gatti M., Bianco E., Michelena J., Imhof R., Altmeyer M. (2019). Phase separation of 53BP1 determines liquid-like behavior of DNA repair compartments. EMBO J..

[87] Michelini F., Pitchiaya S., Vitelli V., Sharma S., Gioia U., Pessina F., Cabrini M., Wang Y., Capozzo I., Iannelli F. (2017). Damage-induced lncRNAs control the DNA damage response through interaction with DDRNAs at individual double-strand breaks. Nat. Cell Biol..

[88] Sharma S., Anand R., Zhang X., Francia S., Michelini F., Galbiati A., Williams H., Ronato D.A., Masson J.Y., Rothenberg E. (2021). MRE11-RAD50-NBS1 complex is sufficient to promote transcription by RNA polymerase II at double-strand breaks by melting DNA ends. Cell Rep..

[89] Ohle C., Tesorero R., Schermann G., Dobrev N., Sinning I., Fischer T. (2016). Transient RNA-DNA hybrids are required for efficient double-strand break repair. Cell.

[90] Yasuhara T., Kato R., Hagiwara Y., Shiotani B., Yamauchi M., Nakada S., Shibata A., Miyagawa K. (2018). Human Rad52 promotes XPG-mediated R-loop processing to initiate transcription-associated homologous recombination repair. Cell.

[91] Cohen S., Puget N., Lin Y.L., Clouaire T., Aguirrebengoa M., Rocher V., Pasero P., Canitrot Y., Legube G. (2018). Senataxin resolves RNA:DNA hybrids forming at DNA double-strand breaks to prevent translocations. Nat. Commun..

[92] D’Alessandro G., Whelan D.R., Howard S.M., Vitelli V., Renaudin X., Adamowicz M., Iannelli F., Jones-Weinert C.W., Lee M., Matti V. (2018). BRCA2 controls DNA:RNA hybrid level at DSBs by mediating RNase H2 recruitment. Nat. Commun..

[93] Aguilera A., Gomez-Gonzalez B. (2017). DNA-RNA hybrids: The risks of DNA breakage during transcription. NSMB.

[94] Marnef A., Legube G. (2021). R-loops as Janus-faced modulators of DNA repair. Nat. Cell Biol..

[95] Rawal C.C., Zardoni L., Di Terlizzi M., Galati E., Brambati A., Lazzaro F., Liberi G., Pellicioli A. (2020). Senataxin ortholog Sen1 limits DNA:RNA hybrid accumulation at DNA double-strand breaks to control end resection and repair fidelity. Cell Rep..

[96] Ortega P., Mérida-Cerro J.A., Rondón A.G., Gómez-González B., Aguilera A. (2021). DNA-RNA hybrids at DSBs interfere with repair by homologous recombination. Elife.

[97] Schwanhäusser B., Busse D., Li N., Dittmar G., Schuchhardt J., Wolf J., Chen W., Selbach M. (2011). Global quantification of mammalian gene expression control. Nature.

[98] Dar R.D., Razooky B.S., Singh A., Trimeloni T.V., McCollum J.M., Cox C.D., Simpson M.L., Weinberger L.S. (2012). Transcriptional burst frequency and burst size are equally modulated across the human genome. Proc. Natl. Acad. Sci. USA.

[99] Suter D.M., Molina N., Gatfield D., Schneider K., Schibler U., Naef F. (2011). Mammalian genes are transcribed with widely different bursting kinetics. Science.

[100] Pennington K.L., Marr S.K., Chirn G.W., Marr M.T. (2013). 2nd. Holo-TFIID controls the magnitude of a transcription burst and fine-tuning of transcription. Proc. Natl. Acad. Sci. USA.

[101] Nicolas D., Zoller B., Suter D.M., Naef F. (2018). Modulation of transcriptional burst frequency by histone acetylation. Proc. Natl. Acad. Sci. USA.

[102] Piovesan A., Caracausi M., Antonaros F., Pelleri M.C., Vitale L. (2016). GeneBase 1.1: A tool to summarize data from NCBI Gene datasets and its application to an update of human gene statistics. Database.

[103] Fuchs G., Voichek Y., Benjamin S., Gilad S., Amit I., Oren M. (2014). 4sUDRB-seq: Measuring genomewide transcriptional elongation rates and initiation frequencies within cells. Genome Biol..

[104] Jonkers I., Kwak H., Lis J.T. (2014). Genome-wide dynamics of Pol II elongation and its interplay with promoter proximal pausing, chromatin, and exons. Elife.

[105] Veloso A., Kirkconnell K.S., Magnuson B., Biewen B., Paulsen M.T., Wilson T.E., Ljungman M. (2014). Rate of elongation by RNA polymerase II is associated with specific gene features and epigenetic modifications. Genome Res..

[106] He Y., Yan C., Fang J., Inouye C., Tjian R., Ivanov I., Nogales E. (2016). Near-atomic resolution visualization of human transcription promoter opening. Nature.

[107] Plaschka C., Hantsche M., Dienemann C., Burzinski C., Plitzko J., Cramer P. (2016). Transcription initiation complex structures elucidate DNA opening. Nature.

[108] Adelman K., Lis J.T. (2012). Promoter-proximal pausing of RNA polymerase II: Emerging roles in metazoans. Nat. Rev. Genet..

[109] Core L.J., Waterfall J.J., Lis J.T. (2008). Nascent RNA sequencing reveals widespread pausing and divergent initiation at human promoters. Science.

[110] Gilchrist D.A., Nechaev S., Lee C., Ghosh S.K., Collins J.B., Li L., Gilmour D.S., Adelman K. (2008). NELF-mediated stalling of Pol II can enhance gene expression by blocking promoter-proximal nucleosome assembly. Genes Dev..

[111] Gilchrist D.A., Dos Santos G., Fargo D.C., Xie B., Gao Y., Li L., Adelman K. (2010). Pausing of RNA polymerase II disrupts DNA-specified nucleosome organization to enable precise gene regulation. Cell.

[112] Laitem C., Zaborowska J., Isa N.F., Kufs J., Dienstbier M., Murphy S. (2015). CDK9 inhibitors define elongation checkpoints at both ends of RNA polymerase II-transcribed genes. Nat. Struct. Mol. Biol..

[113] Reverón-Gómez N., González-Aguilera C., Stewart-Morgan K.R., Petryk N., Flury V., Graziano S., Johansen J.V., Jakobsen J.S., Alabert C., Groth A. (2019). Accurate recycling of parental histones reproduces the histone modification landscape during DNA replication. Mol. Cell.

[114] Chiarle R., Zhang Y., Frock R.L., Lewis S.M., Molinie B., Ho Y.J., Myers D.R., Choi V.W., Compagno M., Malkin D.J. (2011). Genome-wide translocation sequencing reveals mechanisms of chromosome breaks and rearrangements in B cells. Cell.

[115] Seo J., Kim S.C., Lee H.S., Kim J.K., Shon H.J., Salleh N.L., Desai K.V., Lee J.H., Kang E.S., Kim J.S. (2012). Genome-wide profiles of H2AX and γ-H2AX differentiate endogenous and exogenous DNA damage hotspots in human cells. Nucleic Acids Res..

[116] Yan W.X., Mirzazadeh R., Garnerone S., Scott D., Schneider M.W., Kallas T., Custodio J., Wernersson E., Li Y., Gao L. (2017). BLISS is a versatile and quantitative method for genome-wide profiling of DNA double-strand breaks. Nat. Commun..

[117] Ginno P.A., Lim Y.W., Lott P.L., Korf I., Chédin F. (2013). GC skew at the 5′ and 3′ ends of human genes links R-loop formation to epigenetic regulation and transcription termination. Genome Res..

[118] Chen P.B., Chen H.V., Acharya D., Rando O.J., Fazzio T.G. (2015). R-loops regulate promoter-proximal chromatin architecture and cellular differentiation. Nat. Struct. Mol. Biol..

[119] Tan-Wong S.M., Dhir S., Proudfoot N.J. (2019). R-Loops Promote Antisense Transcription across the Mammalian Genome. Mol. Cell.

[120] Sanz L.A., Hartono S.R., Lim Y.W., Steyaert S., Rajpurkar A., Ginno P.A., Xu X., Chédin F. (2016). Prevalent, dynamic, and conserved R-Loop structures associate with specific epigenomic signatures in mammals. Mol. Cell.

